# Management of Microcomplications of Diabetes Mellitus: Challenges, Current Trends, and Future Perspectives in Treatment

**DOI:** 10.3390/biomedicines12091958

**Published:** 2024-08-28

**Authors:** Hande Yapislar, Esra Bihter Gurler

**Affiliations:** 1 Department of Physiology, Faculty of Medicine, Acibadem University, 34752 Istanbul, Türkiye; 2 Department of Basic Sciences, Faculty of Dentistry, Istanbul Galata University, 34430 Istanbul, Türkiye; esrabihter.gurler@galata.edu.tr

**Keywords:** diabetes mellitus, type 2 diabetes mellitus, insulin resistance, hyperglycemia, diabetic retinopathy, diabetic nephropathy, diabetic neuropathy, microvascular complications

## Abstract

Diabetes mellitus is a chronic metabolic disorder characterized by high blood sugar levels, which can lead to severe health issues if not managed effectively. Recent statistics indicate a significant global impact, with 463 million adults diagnosed worldwide and this projected to rise to 700 million by 2045. Type 1 diabetes is an autoimmune disorder where the immune system attacks pancreatic beta cells, reducing insulin production. Type 2 diabetes is primarily due to insulin resistance. Both types of diabetes are linked to severe microvascular and macrovascular complications if unmanaged. Microvascular complications, such as diabetic retinopathy, nephropathy, and neuropathy, result from damage to small blood vessels and can lead to organ and tissue dysfunction. Chronic hyperglycemia plays a central role in the onset of these complications, with prolonged high blood sugar levels causing extensive vascular damage. The emerging treatments and current research focus on various aspects, from insulin resistance to the intricate cellular damage induced by glucose toxicity. Understanding and intervening in these pathways are critical for developing effective treatments and managing diabetes long term. Furthermore, ongoing health initiatives, such as increasing awareness, encouraging early detection, and improving treatments, are in place to manage diabetes globally and mitigate its impact on health and society. These initiatives are a testament to the collective effort to combat this global health challenge.

## 1. Introduction

Diabetes mellitus is a persistent metabolic condition marked by elevated blood sugar levels, which, if not properly managed, can result in severe health issues. It generally occurs from either a lack of adequate insulin production by the pancreas or the body’s inefficiency in utilizing the insulin it generates [1]. According to recent World Health Organization (WHO) statistics, diabetes is a primary global health concern. An estimated 463 million adults (aged 20–79) worldwide are living with diabetes, making up about 9.3% of the global adult population. This prevalence is expected to increase in the coming years. By 2045, the International Diabetes Federation (IDF) projects the number of adults with diabetes to reach 700 million, indicating a substantial rise. Globally, the prevalence of diabetes is relatively balanced between men and women, though the proportion can vary by country. Diabetes prevalence varies significantly across regions and countries. The Middle East and North Africa region has one of the highest diabetes prevalence rates, at 12.2%. In North America and the Caribbean, diabetes affects around 11.1% of the adult population. In Southeast Asia, the prevalence rate is lower, at 8.5%, but due to the large population, this region accounts for many global diabetes cases. Various factors, including lifestyle, diet, genetics, and socioeconomic status, influence the prevalence and incidence of diabetes. For example, urbanization, sedentary lifestyles, and dietary shifts toward processed foods contribute to rising diabetes rates in many regions. Diabetes is a global health issue with diverse regional, gender, and socioeconomic disparities. Ongoing health initiatives aim to address this rising concern, promoting awareness, early detection, and treatment to manage and mitigate its impact [1,2,3].

The different categories of diabetes according to the American Diabetes Association (ADA) are type 1 diabetes mellitus (T1DM), type 2 diabetes mellitus (T2DM), gestational diabetes mellitus (GDM), and other causes of diabetes (such as maturity-onset diabetes of the young (MODY), diseases of the exocrine pancreas, and drug- or chemical-induced diabetes). Type 1 diabetes mellitus, commonly known as T1DM, is an autoimmune disorder in which the immune system targets and eliminates the beta cells in the pancreas that produce insulin. As a result, the pancreas fails to produce enough insulin, an essential hormone that facilitates the entry of glucose (sugar) into cells for energy production. Without sufficient insulin, glucose accumulates in the bloodstream, leading to elevated blood sugar levels. T1DM was formerly known as insulin-dependent diabetes mellitus (IDDM) or juvenile diabetes due to its typical onset in childhood. However, it is now recognized that T1DM can occur at any age [4]. Type 2 diabetes mellitus (T2DM) is a chronic metabolic disorder that leads to elevated blood glucose levels, primarily due to the body’s inefficiency in using insulin. It is closely linked to insulin resistance, a condition where body cells fail to respond adequately to insulin, eventually leading to insufficient insulin production for the body’s needs. Over time, insulin resistance can lead to increased insulin production as the body attempts to overcome this resistance, which may strain the pancreas and eventually contribute to the development of T2DM. While it typically affects adults, it can also emerge in children and adolescents. The incidence of T2DM in children and adolescents is increasing. A study demonstrates the growing prevalence of T2DM in the pediatric population, correlating it with rising childhood obesity rates. T2DM usually progresses slowly and may be controllable through lifestyle adjustments alongside medications. Factors contributing to insulin resistance include obesity, sedentary lifestyles, poor diet, and genetic predisposition [1,5]. T2DM is a significant global health issue, representing nearly 90% of the 537 million cases of diabetes worldwide. The incidence of T2DM is rising rapidly, particularly among children and young adults up to 40 years old. This alarming trend emphasizes the need for early detection and proactive management to prevent and mitigate the risks associated with microvascular and macrovascular complications and reduce the overall mortality burden. Preventive measures, effective treatment, and lifestyle interventions are crucial in managing this condition and curbing its impact on health and society [6,7].

Reports also mention type 3 diabetes, a potential new type of diabetes that has not yet been included in the ADA classification and may have a connection to the brain. T3D describes the connection between insulin resistance or signaling dysfunction and Alzheimer’s disease (AD). T3D manifests as insulin resistance within the brain and impacts neurocognition, contributing to the etiology of AD. Bad insulin signaling in the brain marks T3D, leading to issues associated with rising insulin resistance, the buildup of neurotoxins, neuronal stress, and neurodegeneration [8]. T3D is a metabolic syndrome affecting glucose homeostasis, with mechanisms involving brain insulin resistance and intracellular glucose metabolic disturbances. The relationship between T2DM and AD, brain insulin resistance, tau hyperphosphorylation, and Aβ plaque formation is discussed in T3D [9]. 

Prolonged high blood glucose levels in diabetes mellitus play a vital role in the development of microvascular and macrovascular complications in diabetic patients. Macrovascular complications in diabetes mellitus include conditions affecting the larger blood vessels, such as coronary artery disease, peripheral artery disease, and cerebrovascular disease, which can lead to heart attacks, strokes, and other severe conditions. Atherosclerotic narrowing of large blood vessels increases the risk of cardiovascular events such as heart attacks and strokes, which are major macrovascular complications associated with diabetes mellitus [10,11].

Microvascular complications in diabetes occur in the small blood vessels throughout the body. When sustained over a long period, elevated blood glucose levels can lead to the deterioration of the complex network of small blood vessels. Damage to these small blood vessels can result in reduced blood flow, which subsequently impairs the function of the organs and tissues that these vessels supply. Microvascular complications in diabetes can cause significant pathological and functional changes in various organs and tissues. The microvascular complications of diabetes are most commonly identified as diabetic retinopathy, nephropathy, and neuropathy [12,13].

Chronic hyperglycemia, which refers to long-term high blood sugar levels, is a primary causative factor in the development of microvascular complications in diabetes. Additionally, glycated hemoglobin (HbA1c) has been established as a crucial marker for the microvascular complications of diabetes. HbA1c levels provide a long-term snapshot of an individual’s average blood glucose control over approximately two to three months, making it an essential tool in predicting the risk of developing complications such as neuropathy, nephropathy, and retinopathy. Managing HbA1c levels is vital in reducing the likelihood of these microvascular complications, highlighting the importance of maintaining reasonable blood glucose control [14,15]. This review focuses on the current knowledge of the pathophysiology and treatment of microvascular complications of diabetes and the potential implications on therapy. 

## 2. Role of Metabolic Pathway in Microvascular Complications

Brownlee’s hypothesis, also known as the “metabolic hypothesis”, proposed by Dr. Michael Brownlee, offers a comprehensive explanation for the cellular and molecular mechanisms leading to diabetic complications, both microvascular and macrovascular ones. The hypothesis is centered around the concept of “glucose toxicity” (or “glucotoxicity”) and the overproduction of reactive oxygen species (ROS) in the mitochondria as a response to elevated glucose levels.

The hypothesis begins with the assertion that the persistent elevation of blood glucose levels in diabetes mellitus leads to increased glucose flux through metabolic pathways, culminating in the excessive generation of specific metabolic intermediates. According to Brownlee, diabetes specifically targets endothelial and mesangial cells, whose glucose transport rates remain relatively stable despite hyperglycemia, resulting in elevated intracellular glucose levels. This is important for understanding the underlying mechanism of microvascular complications [16,17].

The elevated generation of ROS is observed in every cell type through the mitochondrial electron transport chain, affected by hyperglycemia-induced damage. This excessive production of ROS is suggested to be the primary and pivotal factor driving the four principal biochemical pathways accountable for the observed damage in diabetes [18].

The polyol pathway plays a significant role in developing the microvascular complications associated with diabetes, including retinopathy, nephropathy, and neuropathy. In hyperglycemia, more glucose is shunted into the polyol pathway. In this pathway, glucose is first reduced to sorbitol by the enzyme aldose reductase, using NADPH as a cofactor. Sorbitol is then metabolized to fructose by sorbitol dehydrogenase, using NAD^+^ as a cofactor. The transformation of glucose into sorbitol utilizes NADPH, an essential coenzyme vital for replenishing glutathione, a paramount antioxidant in cells. Diminishing NADPH levels reduces the cell’s ability to counteract ROS, thus heightening oxidative stress. Sorbitol, generated from the aldose reductase reaction, may amass within cells due to its reduced ability to diffuse across cell membranes compared to glucose. This buildup can induce osmotic stress, resulting in cellular swelling, impairment, and eventual harm. Microvessels, such as those found in the retina, kidneys, and nerves, exhibit heightened vulnerability to the impacts of sorbitol buildup and elevated oxidative stress. In the retina, for instance, these mechanisms may result in leakage from capillaries, the thickening of the basement membrane, and, ultimately, the onset of retinal ischemia and neovascularization, hallmark features of diabetic retinopathy. The activation of the polyol pathway, alongside oxidative stress and other alterations it induces, can promote inflammation, causing additional harm to microvascular architectures. The inflammatory cytokines and growth factors discharged as a reaction to elevated glucose levels intensify vascular permeability and fibrosis. In nerves, the increased polyol pathway flux can lead to reduced nerve conduction velocity and nerve blood flow, contributing to glomerular hyperfiltration and eventually diabetic neuropathy [19,20,21].

Advanced glycation end-products (AGEs) are detrimental substances created when proteins or lipids undergo non-enzymatic glycation due to sugar exposure. In diabetes mellitus, increased blood glucose levels expedite AGE formation, a pivotal factor in the onset of microvascular complications. 

AGEs can modify proteins by means of cross-linking, which changes their configuration and function. This modification impairs protein function and is particularly detrimental in the matrix of blood vessels and within cells like endothelial cells, which line the blood vessels. Proteins that are cross-linked exhibit resistance to degradation, resulting in the accumulation of damaged proteins and changes in cellular and tissue composition [22,23].

The interaction of AGEs with their receptor, RAGE (Receptor for Advanced Glycation End-products), situated on cell surfaces, triggers ROS generation and inflammatory signaling pathways. An increase in oxidative stress and inflammation contributes to vascular damage. AGEs contribute to endothelial dysfunction by impairing endothelial nitric oxide (NO) production. NO is crucial for vascular relaxation and maintaining blood flow. NO modulates vascular tone to enhance the delivery of oxygen. Reduced NO levels lead to increased vascular stiffness, reduced elasticity, and impaired blood flow, exacerbating hypoxia and ischemic tissue damage [24].

**Figure 1:**
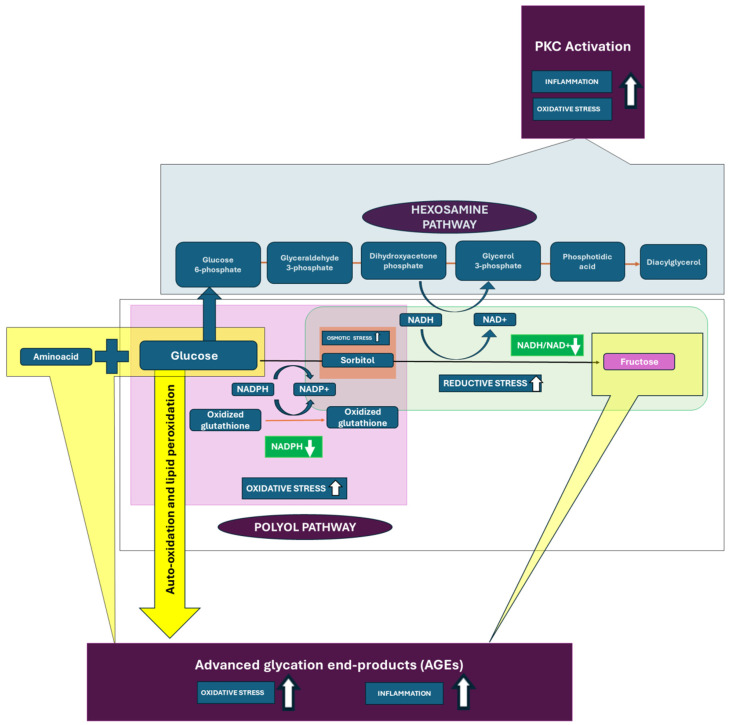
Pathways involved in chronic hyperglycemia-induced cellular damage in diabetes. Each pathway contributes to a cycle of oxidative stress, inflammation, and cellular dysfunction, driving the progression of diabetic microvascular complications such as retinopathy, nephropathy, and neuropathy.

AGEs disrupt the permeability of blood vessels by altering the basement membrane and decreasing the integrity of tight junctions between endothelial cells. Increased permeability can result in the leakage of proteins (such as albumin) and fluids into tissues, thereby playing a role in the development of conditions such as diabetic retinopathy and nephropathy (Figure 1) [22,23].

In the retina, AGEs can stimulate the expression of vascular endothelial growth factor (VEGF), identified as the critical angiogenic molecule promoting abnormal blood vessel growth. VEGF causes increased permeability and increases leakiness and retinal hemorrhage [25]. The accumulation of AGEs is associated with glomerular hyperfiltration, thickening of the glomerular basement membrane, glomerulosclerosis, and tubulointerstitial fibrosis, and, eventually, with the structural changes characteristic of diabetic nephropathy [26].

Protein Kinase C (PKC) is a family of serine/threonine kinases that play pivotal roles in numerous cellular processes. In diabetes mellitus, hyperglycemia stimulates the synthesis of diacylglycerol (DAG) from glycolytic intermediates. DAG is a critical second messenger. The increase in DAG levels leads to the activation of various PKC isoforms such as PKC-α, PKC-β, PKC- PKC-δ, and so on. Their activation profile can vary depending on the cell type and the pathological state. PKC can be activated either directly by the formation of DAG induced by hyperglycemia or indirectly via the polyol pathway. In diabetic complications, PKC-β is often prominently involved, particularly in the vascular tissues. In diabetes, vascular tissues exhibit increased PKC activity, which ultimately leads to increased permeability and dysfunction [26,27,28].

The activation of PKC can phosphorylate a wide range of target proteins that are involved in numerous cellular functions. PKC influences VEGF production in the microvasculature, increasing blood vessel permeability and angiogenesis [27]. In terms of the microvascular complications of diabetes, an increase in PKC activation contributes to increased vascular permeability and neovascularization in the retina, leads to alterations in glomerular filtration and boosts the production of extracellular matrix proteins in the kidney, and affects nerve function directly by altering neuronal signaling and survival, as well as through its impact on blood vessels [27,28].

Glycose is metabolized through a metabolic pathway. However, excess glucose is partly shunted into the hexosamine pathway during hyperglycemia. Fructose-6-phosphate amidotransferase uses fructose-6-phosphate and glutamine to produce glucosamine-6-phosphate, initiating the path. Glucosamine-6-phosphate is further processed to produce UDP-N-acetylglucosamine (UDP-GlcNAc). UDP-GlcNAc is a substrate for various glycosylation reactions within cells that are crucial for modifying proteins and lipids, influencing their function and stability. One of the critical modifications mediated by UDP-GlcNAc is O-GlcNAcylation, where N-acetylglucosamine is added to serine or threonine residues on nuclear and cytoplasmic proteins.

Increased O-GlcNAcylation significantly affects cellular signaling and transcriptional regulation, particularly impacting transcription factors like NF-κB and signaling proteins such as insulin receptor substrate-1 (IRS-1). NF-κB is a crucial transcription factor regulating the genes responsible for immune and inflammatory responses. O-GlcNAcylation can directly modify NF-κB, influencing its nuclear localization and DNA-binding activity. IRS-1 is a critical component of the insulin signaling pathway, which mediates the effects of insulin on glucose uptake and metabolism. The phosphorylation of IRS-1 is essential for its ability to propagate insulin signal transduction; however, O-GlcNAcylation can competitively inhibit this phosphorylation. Alterations in these pathways can exacerbate insulin resistance and inflammatory states, contributing to the pathogenesis of diabetes complications.

Elevated O-GlcNAcylation can impair the function of retinal cells and promote the expression of inflammatory cytokines and growth factors that lead to blood–retina barrier breakdown and the dysfunction of retinal cells. It can also impair the function of cells in the glomerulus and tubules, leading to fibrosis and altering filtration capabilities, which may result in nephropathy. Additionally, increased O-GlcNAcylation can interfere with normal signaling pathways, promoting neuronal damage or death and thereby contributing to diabetic neuropathy.

These pathways collectively lead to oxidative stress, inflammation, and cellular dysfunction, which are critical factors in the development of diabetic complications. Dr. Michael Brownlee’s “metabolic hypothesis” provides a foundational framework for understanding the cellular and molecular mechanisms underlying diabetic complications. While this hypothesis significantly advances our understanding of the biochemical underpinnings of diabetic complications, particularly highlighting the critical role of mitochondrial ROS, it does not encompass all of the variables and complexities of diabetes. Genetic predispositions, differences in individual metabolism, and the presence of other co-morbid conditions are also crucial to the development and progression of complications, underscoring that this hypothesis is part of a larger picture that includes multiple biochemical, genetic, and environmental factors [29,30].

## 3. New Molecular Pathogenesis

### 3.1. Epigenetics Mechanisms

Epigenetics refers to heritable changes in gene expression that do not involve changes to the underlying DNA sequence. In diabetes mellitus, epigenetic modifications play a significant role in developing and progressing microvascular complications by affecting critical biological pathways and gene expressions related to inflammation, endothelial function, and oxidative stress. These complications are often attributed to the persistent effects of hyperglycemia on various cellular processes, even after glucose levels have been normalized, a phenomenon known as “metabolic memory” [16]. The fundamental epigenetic mechanisms are listed below.

DNA methylation is one of the most studied epigenetic modifications in the context of diabetes and its vascular complications. This adds a methyl group to the DNA, typically at cytosine dinucleotides, which generally leads to gene silencing. In diabetes, hyperglycemia induces changes in the DNA methylation pattern of genes involved in inflammatory processes, endothelial function, and oxidative stress. For example, hypermethylation of the promoter region of the endothelial nitric oxide synthase (eNOS) gene has been linked to decreased eNOS expression, contributing to endothelial dysfunction in diabetic vasculopathy [31].

Histone modifications, such as acetylation, methylation, phosphorylation, and ubiquitination, can affect chromatin structure and gene expression, activating or repressing gene transcription. In diabetic patients, increased histone acetylation and decreased histone deacetylation at inflammatory gene promoters have been observed, leading to the overexpression of pro-inflammatory cytokines, which exacerbate microvascular damage, highlighting how changes in histone marks are associated with genes regulating inflammation and cellular stress responses. For example, persistent histone acetylation at the promoters of pro-inflammatory genes such as TNF-α and IL-6 has been reported in endothelial cells exposed to high glucose levels. This phenomenon contributes to a pro-inflammatory state that promotes microvascular damage [32].

miRNAs are small non-coding RNAs that regulate gene expression by targeting mRNA for degradation or translational repression. In diabetes, altered miRNA expression affects genes involved in vascular homeostasis and remodeling, contributing to the pathogenesis of diabetic microvascular complications by affecting endothelial function and fibrosis. For instance, miR-200b and miR-222 are upregulated in endothelial cells under diabetic conditions, leading to increased vascular permeability and endothelial dysfunction [33]. In contrast, miR-192 is upregulated in diabetic nephropathy, promoting collagen expression in the kidney by targeting E-box repressors, leading to fibrosis and kidney function decline [34]. 

Long non-coding RNAs (lncRNAs) and microRNAs (miRNAs) regulate gene expression at various levels, including chromatin modification, miRNA sequestration, and post-transcriptional control, and their dysregulation has been implicated in diabetic complications. For example, the long non-coding RNA MALAT1 (metastasis-associated lung adenocarcinoma transcript 1) is upregulated in models of diabetes, promoting VEGF expression and contributing to diabetic retinopathy by inducing retinal endothelial cell dysfunction, a key event in the development of this complication [35]. Specific miRNAs, such as miR-126, are known to be downregulated in diabetes, which negatively affects their usual role in maintaining endothelial function and vascular integrity [36]. 

**Figure 2:**
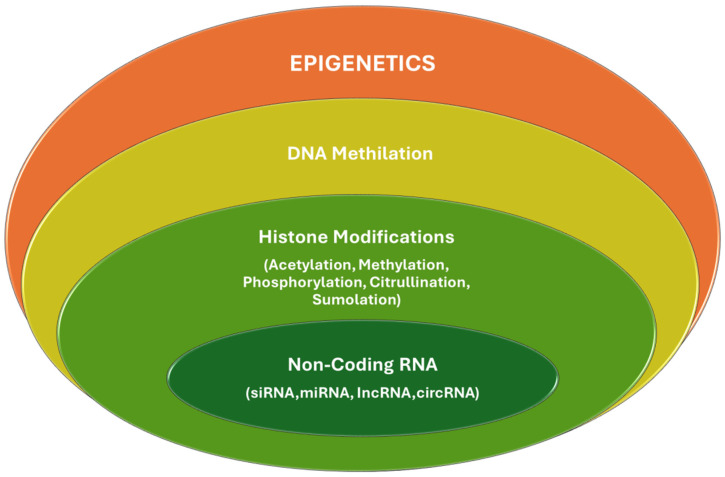
Layers of epigenetic regulation. This diagram illustrates the hierarchical structure of epigenetic regulation mechanisms. The layers represent different levels of epigenetic modifications that regulate gene expression without altering the DNA sequence. Epigenetics: The outermost layer encompasses all epigenetic mechanisms that influence gene expression and cellular function. DNA Methylation: This layer involves the addition of methyl groups to DNA, typically at CpG sites, leading to gene silencing or activation depending on the context. Histone Modifications: This layer includes various chemical modifications to histone proteins, such as acetylation, methylation, phosphorylation, citrullination, and sumoylation. These modifications alter chromatin structure and influence gene expression by either promoting or inhibiting the accessibility of transcriptional machinery to DNA. Non-Coding RNA: The innermost layer consists of non-coding RNAs, including small interfering RNAs (siRNAs), microRNAs (miRNAs), long non-coding RNAs (lncRNAs), and circular RNAs (circRNAs). These RNA molecules regulate gene expression at the transcriptional and post-transcriptional levels through various mechanisms, such as RNA interference and modulation of chromatin structure. Together, these layers represent the complex network of epigenetic regulation that modulates gene activity and contributes to cellular differentiation, development, and disease processes.

Epigenetic changes in diabetes are complex and contribute significantly to the vascular complications associated with the disease by affecting key pathways in endothelial function, inflammation, and extracellular matrix remodeling. Understanding these changes offers potential therapeutic targets for managing or preventing these complications, emphasizing the importance of reasonable glycemic control. Targeting these epigenetic modifications presents a promising avenue for developing new therapeutic strategies to address these debilitating conditions (Figure 2).

### 3.2. Role of Gut Microbiota 

The gut microbiota plays a significant role in human health, impacting physiological processes from metabolism to immune function. In the context of diabetes, the composition and function of the gut microbiota have been linked to the pathogenesis and progression of both the disease and its vascular complications. Here is how the gut microbiota influences vascular complications in diabetes:

The gut microbiota can influence systemic inflammation and metabolic control, which is crucial in developing vascular complications. Dysbiosis, a disrupted microbial balance, can lead to increased permeability of the gut barrier (leaky gut), allowing bacterial products such as lipopolysaccharide (LPS) to enter the bloodstream. This triggers low-grade systemic inflammation by activating inflammatory pathways such as NF-κB, which are implicated in vascular damage [37].

Certain gut bacteria can produce metabolites like trimethylamine N-oxide (TMAO), which is linked to atherosclerosis—a major vascular complication in diabetes. TMAO is derived from dietary choline and carnitine, and higher levels are associated with an increased risk of cardiovascular events [38].

The gut microbiota can influence host glucose metabolism through multiple mechanisms, including the modulation of bile acid metabolism, production of short-chain fatty acids (SCFAs) like butyrate and propionate, and hormonal regulation. SCFAs, for example, can improve insulin sensitivity and exert anti-inflammatory effects, potentially reducing the risk of vascular complications [39].

The gut microbiota also regulates the immune system. An imbalanced microbiota can alter immune responses, promoting chronic inflammation, a known contributor to endothelial dysfunction and vascular complications in diabetes [40].

As a result, the relationship between the gut microbiota and vascular complications in diabetes is complex and mediated through various mechanisms, including metabolic regulation, inflammation, immune modulation, and production of specific metabolites. Targeting the gut microbiota through dietary changes, probiotics, or other microbiome-modulating therapies holds potential for preventing or managing vascular complications in diabetes, highlighting the importance of this emerging field of research.

### 3.3. Diabetic Retinopathy 

Diabetic retinopathy (DR) is a complication of diabetes mellitus characterized by damage to the blood vessels of the retina, the light-sensitive tissue at the back of the eye. It can lead to vision impairment and is classified into two main stages: nonproliferative diabetic retinopathy (NPDR) and proliferative diabetic retinopathy (PDR) [41,42]. NPDR, the initial stage, features increased vascular permeability and capillary occlusion, which can lead to retinal pathologies without neovascularization [42]. PDR, the more advanced stage, involves the growth of new, fragile blood vessels on the retina and into the vitreous humor, potentially causing complications such as vitreous hemorrhage and retinal detachment, which may lead to blindness [42,43].

Approximately one-third of individuals with diabetes have some form of DR, and a third of these individuals may have a form of the disease that threatens their vision, whether it be severe NPDR, PDR, or diabetic macular edema [41]. Diabetic macular edema, another complication, involves fluid accumulation in the macula, the central part of the retina, leading to vision loss [23]. Regular screening is imperative to assess the presence and progression of DR, as early detection and treatment are crucial to managing the condition and preventing severe visual impairment [23]. Clinical findings such as hemorrhages and cotton wool spots, along with diagnostics from fundus fluorescein angiography (FFA), optical coherence tomography (OCT), and OCT-angiography (OCT-A), are instrumental in detecting and monitoring DR. Diabetic retinopathy is cited as a significant cause of vision loss in working-age populations, emphasizing the need for better management of diabetes and regular ophthalmic examinations [42].

The management of DR includes controlling systemic factors like blood glucose and hypertension and treatments such as anti-VEGF therapy, laser photocoagulation, and, in advanced cases, vitreoretinal surgery [42]. The risk of developing DR correlates with both the duration of diabetes and the level of blood sugar control. The Wisconsin Epidemiologic Study of Diabetic Retinopathy found that the prevalence of DR increases significantly with a younger age at diagnosis, especially when diagnosed before the age of 30 years [44]. Additionally, the UK Prospective Diabetes Study (UKPDS) showed that among patients with T2DM, there are significant risk factors for both the incidence and progression of retinopathy over six years from diagnosis, highlighting the importance of early management to reduce these risks [42].

In T1DM, almost all patients develop some form of retinopathy after 20 years of having the disease. For T2DM, more than 60% of patients develop some form of retinopathy over the same duration. Risk factors for the development of diabetic retinopathy (DR) include hyperglycemia, hypertension, dyslipidemia, obesity, sleep apnea, the duration of diabetes mellitus, smoking, and genetic factors, among others [45]. Moreover, achieving a better control of glycemic levels has been associated with a lower prevalence of DR, as demonstrated in Japan, where more stringent treatment targets led to a decrease in DR prevalence over a decade.

Panretinal photocoagulation and intravitreal injections of ranibizumab are treatment options for proliferative diabetic retinopathy and have been researched for their efficacy and outcomes, broadening the understanding of treatment impacts on the progression and management of proliferative DR [46].

Inflammation plays a vital role in the pathogenesis of DR. It is a primary event in T1DM, where the disease can be initiated by infectious or autoimmune processes, while in T2DM, chronic inflammation results from increasing insulin resistance and disturbed glucose metabolism [47].

Chronic inflammation, in particular, leads to structural and molecular changes in the retina, resulting in tissue damage and cell death [48,49]. Diabetic retinopathy, as a neurodegenerative disease, features the activation of inflammatory pathways and metabolic abnormalities such as the polyol pathway, the hexosamine biosynthetic pathway, the protein kinase C pathway, and the accumulation of AGEs, which contribute to increased oxidative stress and inflammation within the retina [50,51,52].

Chronic low-grade inflammation is present at different stages of diabetic retinopathy. It is implicated in damage to the retinal vasculature, contributing to the two major causes of visual impairment in diabetes: diabetic macular edema and proliferative diabetic retinopathy (PDR) [53,54]. Moreover, diabetes causes an increase in the local and systemic production of numerous inflammatory molecules involved in the development of DR.

Rapid improvements in systemic glucose control can temporarily worsen diabetic retinopathy; however, this emphasizes the complexity of the disease’s relationship with metabolic control and inflammation [55]. Furthermore, photoreceptor cells have been found to produce inflammatory products that contribute to retinal vascular permeability in diabetic conditions [56,57].

The presence and progression of diabetic retinopathy are not only health concerns but also associated with a significant increase in healthcare costs, underlining the importance of preventive care and effective diabetes management [57].

### 3.4. Treatment and Current Advances in Diabetic Retinopathy

Treatment options for DR are advancing, and current treatments focus on preventing the disease’s progression and enhancing visual outcomes. The Early Treatment Diabetic Retinopathy Study (ETDRS) has shown the benefit of focal laser photocoagulation in decreasing the frequency of persistent macular edema and increasing the chance of visual improvement [42]. Panretinal photocoagulation, as per the Diabetic Retinopathy Study (DRS), remains a cornerstone in reducing the rate of severe visual loss in proliferative diabetic retinopathy (PDR).

Current advances in pharmacotherapy and technology include precise control of laser treatments, finding new ways to minimize treatment time, and decreasing collateral damage [42,43,45,58]. However, the destructive nature of the current laser treatments can be associated with adverse effects, which has prompted the search for novel therapies [55,59].

Additionally, diabetes mellitus necessitates long-term management and interventions. Although diabetic retinopathy is mainly asymptomatic, progression to advanced stages can significantly reduce the effectiveness of the treatments available today. Hence, there is a growing need for more efficient strategies to prevent and treat DR in its early stages [60,61]. Intravitreal injections of anti-VEGF agents have become a mainstay for treating severe nonproliferative DR and PDR, often associated with or without focal laser therapy for diabetic macular edema. It is important to note that while these therapies can be effective, they may also have limitations concerning administration comfort, long-term side effects, and costs [43,62]. Therefore, the field is exploring polyphenols like resveratrol for their potential benefits related to oxidative stress, which contributes to DR [43].

Polyphenols are natural compounds with antioxidant properties and have been studied for their potential therapeutic applications in various pathologies, including diabetes and diabetic retinopathy. There is growing evidence supporting the role of polyphenols in preventing and treating diabetic complications [63]. The polyphenols present in certain plants, such as Pterocarpus marsupium and resveratrol, have been shown to have antihyperglycemic effects and improve glucose metabolism [64,65].

Resveratrol, in particular, has been widely studied and has shown potential as a non-toxic agent to prevent the progression of diabetic retinopathy [66]. Other polyphenols that promise to avoid diabetes mellitus-induced retinopathy include green tea polyphenols, curcumin, quercetin, and cocoa polyphenols [65]. Polyphenol-induced NRF2 activation has also been indicated in reducing the oxidative stress on the retina [67]. Resveratrol has been shown to suppress hyperglycemia-mediated inflammation and reduce Cx43 by downregulating VEGF, TGF-β1, and PKC-β, critical regulators for ROS generation [68]. Zeaxanthin, a dietary carotenoid, has been demonstrated to alleviate retinal oxidative stress and inflammatory response in diabetic rats, as well as attenuate VEGF-induced neovascularization in the human retina via the Nox4-dependent pathway [69]. Sulforaphane, a compound found in cruciferous vegetables such as broccoli, kale, and cauliflower, is known for its antioxidant and anti-inflammatory properties and has been studied extensively for its potential health benefits, including its ability to protect against certain types of cancer and support heart health. Sulforaphane induces promoter demethylation and histone acetylation to upregulate the expression of Nrf2, a vital regulator of the oxidative stress response, and affects the protein levels of DNMT1 and DNMT3a [70,71]. Dietary factors such as dietary fiber, a low glycemic index, and low-calorie meal intake have been associated with a protective effect on diabetic retinopathy, potentially reducing the risk of its occurrence and progression [41].

Adeno-associated virus (AAV)-mediated injections of Flt23k have been shown to inhibit murine choroidal neovascularization, indicating the potential of targeted anti-VEGF therapies for diabetic retinopathy [44].

These insights collectively provide a multifaceted understanding of angiogenic drugs’ potential mechanisms, therapeutic implications, and related interventions in diabetic retinopathy. Further research and clinical investigations are warranted to elucidate these approaches’ specific mechanisms and efficacy in managing diabetic retinopathy.

The role of vitamin D in diabetic retinopathy has been a subject of interest in the context of oxidative stress and inflammatory processes. Several studies have shown that vitamin D protects against oxidative stress in diabetic retinopathy by increasing cellular glutathione, regulating the redox state, and enhancing the formation of Sirt1 to improve mitochondrial function [72,73]. Kaštelan et al. have highlighted the significance of VEGF in the pathogenesis of diabetic retinopathy, indicating a correlation between the VEGF levels in the serum and vitreous with diabetic retinopathy and diabetic macular edema. Research on diabetic peripheral neuropathy also points towards its role in supporting nerve health, which could be indirectly associated with the mechanisms influenced by VEGF, as VEGF is known to play a critical role in angiogenesis and vascular function [74]. Recent in vitro experiments have discovered that calcitriol, the active form of vitamin D, can hinder the growth of new blood vessels by decreasing VEGF levels [75]. This suggests a potential link between angiogenic factors and the progression of diabetic retinopathy, which may be influenced by vitamin D levels [45]. Moreover, the protective role of vitamin D against oxidative stress in diabetic retinopathy indicates its potential to regulate mitochondrial function and cellular bioenergetics, as well as its effects on the immune system and angiogenesis. This underscores the multifaceted impact of vitamin D on the pathophysiological mechanisms associated with diabetic retinopathy [72]. Furthermore, the potential ameliorative effects of orally administered antioxidants containing vitamin D3, in conjunction with other components such as α-lipoic acid and genistein, in alleviating the electroretinogram oscillatory potential in patients with diabetic retinopathy have been demonstrated. This suggests a potential role for vitamin D as part of a multi-component nutritional formula in addressing the functional impairments associated with diabetic retinopathy [68].

Overall, the potential role of vitamin D in mitigating oxidative stress, regulating angiogenic factors, and influencing retinal function underscores its significance in diabetic retinopathy. Further research and clinical investigations are warranted to elucidate vitamin D’s specific mechanisms and therapeutic implications in managing diabetic retinopathy.

The loss of retinal pericytes is one of the earliest changes in DR, and recent studies have reported that hyperglycemia-induced cellular stress stimulates the pyroptosis of retinal microglia through the NLRP3 inflammasome signaling pathway [76]. Pyroptosis is a form of programmed cell death characterized by a highly inflammatory response. Specialized cell death occurs in response to infection or cellular stress. The activation of inflammasomes primarily mediates pyroptosis. Upon activation, inflammasomes recruit and activate caspase-1, which cleaves pro-inflammatory cytokines. Additionally, caspase-1 cleaves Gasdermin D (GSDMD), forming GSDMD-N, which forms pores in the cell membrane. These pores cause cell swelling, membrane rupture, and the release of pro-inflammatory molecules, including IL-1β, IL-18, and DAMPs, into the extracellular space. Pyroptosis is critical in host defense against microbial infections by eliminating infected cells and activating the immune response. However, dysregulated or excessive pyroptosis can contribute to the pathogenesis of various inflammatory diseases. Recent studies have reported that hyperglycemia-induced cellular stress stimulates pyroptosis and that different signaling pathways play crucial roles in regulating pyroptosis [77]. Taken together, these findings suggest a strong relationship between oxidative stress, chronic inflammation, and pyroptosis in the pathogenesis of all diabetic microvascular complications (DMCs).

Moreover, the long noncoding RNA KCNQ1OT1 has been found to induce pyroptosis in diabetic corneal endothelial keratopathy [78]. According to past reports, the human genome is transcribed, but only 2% of it codes for proteins. The vast majority of the genome is transcribed as non-coding RNAs, including many subtypes. These non-coding RNAs are arbitrarily divided into two major categories based on their size: small non-coding RNAs and lncRNAs. Non-coding RNAs containing more than 200 nucleotides are defined as lncRNAs and play critical roles in diabetes and diabetic complications [79]. LncRNAs have been implicated in regulating hepatic glucose production and insulin resistance, significant factors contributing to T2DM. For example, lncRNA MEG3 has been shown to increase gluconeogenesis and impair insulin-stimulated glycogen synthesis, promoting hepatic insulin resistance [80]. LncRNA Gomafu and lncRNA MEG3 also contribute to the inappropriate activation of gluconeogenesis. They have also been found to regulate the function of islet β cells responsible for insulin synthesis and secretion. LncRNAs such as MEG3, PLUTO, and H19 have been identified as regulators of insulin synthesis and secretion in pancreatic β cells [81,82]. Dysregulation of these lncRNAs can lead to impaired β cell function and contribute to the pathogenesis of diabetes. LncRNAs can regulate gene expression through epigenetic mechanisms, such as DNA methylation, histone modifications, and chromatin remodeling. Dysregulation of these epigenetic processes has been implicated in the development of diabetes. LncRNAs can interact with chromatin-modifying complexes and transcription factors to regulate gene expression in a cell- and tissue-specific manner.

LncRNAs have also been implicated in the development of DR, which is a vision-threatening complication of diabetes. They are involved in processes such as aberrant neovascularization and neuronal dysfunction in the retina. LncRNAs such as ANRIL, MIAT, and MALAT1 have been found to regulate angiogenesis, vascular permeability, and inflammatory responses in the retina [79]. These findings suggest that pyroptosis may play a role in the pathogenesis of DR. However, the specific inhibitors of pyroptosis in retinal cells have not been well studied and further investigation is needed to fully understand the relationship between pyroptosis and DR. Nonetheless, the inhibition of excessive pyroptosis has emerged as a promising strategy for the treatment of diabetic retinopathy.

These potential therapeutic approaches collectively offer diverse strategies for addressing diabetic neuropathy in the retina, emphasizing the multifaceted nature of interventions for managing this condition.

#### Diabetic Neuropathy

Diabetic neuropathy is a condition characterized by the symptoms and signs of peripheral nerve dysfunction in patients with diabetes [83]. It is a common complication of both T1DM and T2DM. Diabetic neuropathy can affect various nerves, including those in the peripheral, autonomic, and cranial nerves [84]. It is characterized by pain, numbness, tingling, and weakness in the affected areas [85]. The exact cause of diabetic neuropathy is not fully understood, but it is believed to be related to high blood sugar levels, metabolic imbalances, and oxidative stress [86]. The treatment for diabetic neuropathy focuses on managing symptoms, controlling blood sugar levels, and preventing further nerve damage [87].

There are several types of diabetic neuropathy, each affecting different nerves in the body. Autonomic neuropathy affects the nerves that control involuntary bodily functions, such as digestion, heart rate, blood pressure, and bladder function. Symptoms can vary depending on which organs or systems are affected. Common symptoms include digestive problems (such as gastroparesis), cardiovascular issues (such as abnormal heart rates or blood pressure), bladder dysfunction, and sexual dysfunction [84]. 

Proximal neuropathy, also known as diabetic amyotrophy or diabetic lumbosacral radiculoplexus neuropathy, affects the nerves in the thighs, hips, buttocks, and legs. It typically affects one side of the body and can cause severe pain, muscle weakness, and difficulty with movement [84]. Focal neuropathy, also known as mononeuropathy, affects a specific nerve or group of nerves, often resulting in sudden and severe symptoms. It can affect any body part, such as the head, torso, or limbs. Symptoms may include pain, weakness, and muscle wasting in the affected area [84].

Diabetic peripheral neuropathy (DPN) is a widespread complication of diabetes that results in symptoms related to dysfunction in the peripheral nervous system. It affects the peripheral nerves responsible for transmitting signals between the central nervous system and the rest of the body. It is the second leading cause of post-traumatic nerve injury and affects all peripheral nerves, including those responsible for pain sensation, motor function, and the autonomic nervous system. As diabetes becomes more prevalent globally, so does the occurrence of DPN [88,89]. Individuals with DPN typically experience pain in their limbs, particularly toward the extremities, and often describe a sensation similar to wearing gloves and socks [90]. It can also lead to muscle weakness and difficulty walking.

Diabetic polyneuropathy is a specific type of peripheral neuropathy that occurs as a complication of diabetes mellitus. It is characterized by damage to multiple peripheral nerves throughout the body, particularly in the feet and legs. This condition is caused by prolonged exposure to high blood sugar levels, which can lead to nerve damage and dysfunction. Diabetic polyneuropathy can result in a range of symptoms, including numbness, tingling, burning sensations, pain, muscle weakness, and loss of coordination. It often presents in a “stocking and glove” distribution, affecting the extremities first. The severity and progression of diabetic polyneuropathy can vary among individuals, and it can significantly impact quality of life. The management of diabetic polyneuropathy involves controlling blood sugar levels, relieving symptoms, and preventing further nerve damage [91]. 

It is important to note that some individuals may experience a combination of these types of neuropathy and that the severity of symptoms can vary from person to person. The pathophysiology of neuropathy in diabetes is complex and involves multiple mechanisms [92]. Inflammatory processes play a role in the development and progression of diabetic neuropathy [93]. Chronic low-grade inflammation can occur in the nerves, leading to the release of pro-inflammatory molecules and the activation of immune cells. This neuroinflammation can contribute to nerve damage and pain [94,95]. 

Hyperglycemia also activates several biochemical pathways, such as the polyol and hexosamine pathways [84,96], and can damage the small blood vessels that supply nerves, leading to impaired blood flow and reduced oxygen and nutrient supply to the nerves. This microvascular dysfunction can contribute to nerve damage and dysfunction [97]. Diabetes can cause structural changes in nerves, leading to diabetic neuropathy. DM can lead to the loss of nerve fibers, particularly the small nerve fibers responsible for transmitting pain and temperature sensations. This can result in sensory disturbances and loss of sensation in the affected areas. The myelin sheath around nerve fibers can become damaged or destroyed, leading to slowed or disrupted nerve conduction. Diabetes can also cause degeneration or damage to the axons, impairing nerve function and communication [84].

Mitochondrial failure can result in decreased energy generation, elevated reactive oxygen species (ROS) levels, and compromised cellular activity. These factors can also contribute to vascular impairments, such as arteriosclerosis obliterans (ASO). Blood clots are more likely to form in people with diabetes, and reports have shown that AGEs, C-reactive protein, and oxidized low-density lipoproteins cause plaque to break down in the lining of blood vessels by making matrix metalloproteinase-9. All of these factors contribute to the progression of arteriosclerosis, and the occurrence of ASO is approximately 2–4 times greater [98].

The dorsal root ganglia (DRG) neurons play a crucial role in DPN. The DRG neurons are located in the dorsal root ganglia, clusters of nerve cell bodies along the spinal cord [91]. These neurons transmit sensory information from the peripheral nerves to the central nervous system [99,100]. Hyperglycemia and metabolic stressors directly target the sensory neurons in the DRG. This leads to degeneration of the distal axons, which are the long projections of the neurons that transmit signals to peripheral tissues. The preferential involvement of sensory neurons in DPN is responsible for the characteristic sensory alterations experienced by individuals with the condition, such as loss of sensation, impaired balance, and pain in the distal parts of the extremities [101,102]. The dysfunction and degeneration of DRG neurons in DPN contribute to the development of neuropathic symptoms. These neurons transmit pain signals, proprioception (awareness of body position), and other sensory functions. When these neurons are damaged, they can result in the loss of sensation, abnormal sensations (such as tingling or burning), and pain for individuals with DPN [91].

It is important to note that these mechanisms are interconnected and can influence each other, leading to a vicious cycle of nerve damage and dysfunction. The exact contribution of each mechanism may vary among individuals and different types of diabetic neuropathy. Further research is needed to fully understand the pathophysiology of diabetic neuropathy and develop targeted treatments. Understanding the role of DRG neurons in DPN is essential for developing strategies to target and protect these neurons. Various therapeutic approaches, such as gene delivery and modulation of neurotrophic signal transduction pathways, are being explored to promote regeneration and maintain axon integrity in the face of ongoing degeneration in DPN.

### 3.5. Treatment and Current Advances in Diabetic Neuropathy

The current approach to managing diabetic neuropathy involves a combination of strategies to control symptoms, prevent further nerve damage, and improve overall quality of life. Maintaining reasonable blood sugar control is essential in managing diabetic neuropathy. This involves following a healthy diet, monitoring blood sugar levels, taking prescribed medications (such as insulin or oral hypoglycemic agents), and engaging in regular physical activity [84]. However, the effect of glycemic control is controversial for individuals with T2DM [103].

Addressing pain is a crucial aspect of managing diabetic neuropathy. This may involve the use of medications such as anticonvulsants (particularly alpha-2-delta ligands), antidepressants (mainly tricyclics), serotonin–norepinephrine reuptake inhibitors (SNRIs), opiate-receptor agonists, and topical agents like alpha-2-delta ligand (pregabalin) to help alleviate pain symptoms [104,105]. Topical treatments may also be used, such as lidocaine patches or capsaicin creams. In some cases, nerve blocks or spinal cord stimulation may be considered for more severe pain [106].

Other symptoms associated with diabetic neuropathy, such as numbness, tingling, or muscle weakness, can be managed through various approaches. Physical and occupational therapy may be recommended to improve strength, balance, and coordination. Assistive devices, such as braces or orthotics, can help with mobility and reduce the risk of falls.

Proper foot care is crucial in preventing complications related to diabetic neuropathy. Regular foot inspections, adequate hygiene, wearing comfortable and well-fitting shoes, and avoiding injury are essential to reduce the risk of foot ulcers and infections [107]. Adopting a healthy lifestyle can have a positive impact on diabetic neuropathy. This includes maintaining a healthy weight, engaging in regular physical activity, quitting smoking, and limiting alcohol consumption. These lifestyle modifications can help improve blood circulation, reduce inflammation, and promote overall nerve health. Education plays a vital role in managing diabetic neuropathy. Patients should be educated about the condition, its potential complications, and the importance of self-care. This includes regularly monitoring blood sugar levels, adhering to medication regimens, and seeking medical attention for any concerning symptoms [87,108].

Managing diabetic neuropathy often requires a multidisciplinary approach involving healthcare professionals from various specialties. This may include endocrinologists, neurologists, pain specialists, podiatrists, physical therapists, and dietitians. Collaborative care ensures comprehensive management and addresses the diverse aspects of the condition. 

The treatment options for diabetic polyneuropathy also include neurotrophic factors (NGF, GDNF) and stem cell therapy (BM-MNCs, pluripotent stem cells, EPCs, MSCs, DPSCs, ESCs). These options aim to manage symptoms, promote nerve regeneration, and reduce the risk of complications [91]. Some therapeutic strategies for targeting DRG include gene delivery in the context of DPN.

Oligonucleotides are short sequences of nucleic acids that can be designed to target specific genes or gene products. In the case of DPN, oligonucleotide therapeutics can be used to modulate gene expression in DRG neurons. For example, particular molecules such as insulin, GLP-1, PTEN, HSP27, RAGE, CWC22, and DUSP1 that impact neurotrophic signal transduction and other cellular networks may be delivered to DRG neurons using oligonucleotide-based approaches [109,110,111,112].

Antisense oligonucleotides (ASOs) are synthetic molecules that bind to specific RNA sequences and modulate gene expression. In the context of DPN, ASOs can target and inhibit gene expression in developing DRG neurons. By reducing the expression of these genes, it may be possible to protect the neurons and prevent further damage [113].

DNA/RNA heteroduplex oligonucleotides (HDOs) are oligonucleotide therapeutic types that combine DNA and RNA sequences [113]. These molecules can be designed to provide more efficient gene knockdown in DRG neurons compared to single-stranded antisense oligonucleotides. HDOs have shown functional impacts on the development of DPN and provide insights into the regulatory complex that may be pathogenic [91].

LncRNAs have emerged as potential therapeutic targets in neuropathy treatment, including DPN. LncRNAs can regulate gene expression by interacting with DNA, RNA, and proteins [114]. They can act as scaffolds, guides, or decoys to modulate the activity of transcription factors, chromatin modifiers, and other regulatory molecules. By targeting the specific lncRNAs involved in neuropathy, it may be possible to restore standard gene expression patterns and alleviate disease symptoms. Specific lncRNAs have been implicated in promoting neuroprotection and regeneration in the context of neuropathy [115]. For example, MALAT1 (metastasis-associated lung adenocarcinoma transcript 1) has been shown to promote synapse formation and neurite outgrowth in neurons [116,117]. Targeting MALAT1 or other neuroprotective lncRNAs could enhance the survival and function of neurons affected by neuropathy. LncRNAs can play a role in alternative splicing, generating multiple protein isoforms from a single gene. Dysregulation of splicing has been implicated in various neuropathies. By targeting the lncRNAs involved in splicing regulation, correcting aberrant splicing events and restoring normal protein function may be possible. LncRNAs can interact with chromatin and influence epigenetic modifications, such as DNA methylation and histone modifications [118]. These modifications can have long-lasting effects on gene expression. Targeting the lncRNAs involved in epigenetic regulation may provide a means to modulate the gene expression patterns associated with neuropathy.

LncRNAs have a role in future treatment approaches and have shown promise as potential biomarkers for neuropathy [119]. Identifying the specific lncRNAs that are dysregulated in neuropathic conditions could serve as a diagnostic marker for the early detection and monitoring of disease progression. Additionally, lncRNAs could be used as therapeutic targets or indicators of treatment response. It is important to note that lncRNA research is still relatively new, and further studies are needed to fully understand the roles and therapeutic potential of specific lncRNAs in neuropathy. However, targeting lncRNAs holds promise as a novel approach for treating and managing neuropathic conditions.

These therapeutic strategies aim to target and modulate gene expression in DRG neurons to promote regeneration, protect against degeneration, and alleviate the symptoms of DPN. They offer potential avenues for developing novel treatments for this condition.

Stem cell therapy has emerged as a potential treatment option for DPN [105]. Different types of stem cells, including bone marrow-derived cells (BM-MNCs), pluripotent stem cells (PSCs), endothelial progenitor cells (EPCs), mesenchymal stem cells (MSCs), and dental pulp stem cells (DPSCs), have shown promise in promoting nerve regeneration, reducing pain, and improving blood flow in DPN [105,120]. Stem cells exert their therapeutic effects through differentiation, paracrine secretion, immunomodulation, and angiogenesis. Research findings indicate improved nerve conduction velocity, reduced pain and sensory symptoms, and enhanced nerve regeneration in response to stem cell therapy [121]. However, challenges such as tumorigenicity risk, immune rejection, and ethical considerations must be addressed. Further research is needed to optimize protocols, understand long-term effects, and conduct large-scale clinical trials to establish the safety and efficacy of stem cell therapy in DPN.

Exercise interventions have shown promising results in improving the outcomes for individuals with diabetic neuropathy. Different types of exercise interventions, including sensorimotor training, endurance training, strength training, whole-body vibration training, interactive balance training, and proprioceptive exercises, are beneficial. These interventions target various aspects such as balance, muscle strength, vibration perception threshold, gait biomechanics, foot function, and overall quality of life in individuals with diabetic neuropathy. Studies have demonstrated the effectiveness of exercise in improving the sensory and motor symptoms, balance, quality of life, neuropathic pain, nerve conduction velocity, and HbA1c levels in patients with diabetic neuropathy. Endurance and sensorimotor training are recommended as effective interventions for improving balance, glycemic control, nerve conduction velocity, and overall symptoms in patients with diabetic neuropathy [122].

#### Diabetic Nephropathy

Diabetic nephropathy (DN) is a chronic complication of diabetes characterized by high levels of albumin excretion in the urine and declining kidney function [123]. It is a progressive kidney disease caused by damage to the capillaries in the kidney’s glomeruli, which usually filter waste and excess substances from the blood [124,125]. This condition typically arises due to the interaction between metabolic and hemodynamic factors that trigger specific pathways leading to kidney injury. Intensive glucose control has been shown to decrease the progression of DN [123].

The early stages of diabetic nephropathy can present with hyperfiltration due to hyperglycemia-induced vasodilation of the afferent arterioles and can progress to microalbuminuria and macroalbuminuria. As the disease advances, patients may develop hypertension, proteinuria, a reduction in the glomerular filtration rate (GFR), and ultimately end-stage renal disease (ESRD) [126]. Therefore, there was a significant interaction between diabetes and hypertension and between diabetes and increased BMI; however, although increased BMI may be a risk factor for severe Chronic Lung Tissue Ischemia (CLTI) requiring Lower Extremity Amputation (LEA) in those without diabetes, these factors may not have a further effect on LEA risk in the presence of diabetes [127]. Studies have previously proposed a common pathway for diabetes, hypertension, and obesity via the formation of AGEs and increased uptake of low-density lipoproteins into macrophages [128]. In the presence of diabetes, the additional risk factors of hypertension and obesity are no longer conferring further risk.

The pathogenesis of DN involves a complex interplay of various factors that contribute to the structural and functional deterioration of the kidneys in individuals with diabetes. Critical aspects in the development of DN include metabolic and hemodynamic changes, inflammation, and genetic predisposition [123]. The metabolic changes resulting from hyperglycemia lead to the formation of AGEs and the activation of the polyol pathway, which contribute to oxidative stress and tissue damage [128].

Hemodynamic alterations are also significant in the pathogenesis of DN. Initially, diabetes causes hyperfiltration and hypertrophy in the glomeruli due to vasodilation of the afferent arterioles and constriction of the efferent arterioles. The renin–angiotensin system drives these hemodynamic changes and can lead to micro- and macroalbuminuria. Over time, this progresses to proteinuria, hypertension, and a decline in the GFR, potentially culminating in ESRD [129].

Inflammation plays a crucial role in DN. Proinflammatory cytokines such as interleukins and tumor necrosis factor-alpha are elevated in diabetes, which contributes to kidney damage [130,131]. Furthermore, macrophages have been implicated in pathogenesis due to their role in mediating inflammation and fibrosis within the kidney [132,133]. Additionally, genetic factors add to the risk of developing DN. For example, the Haptoglobin 2-2 phenotype is associated with an increased risk of diabetic vascular complications, which includes DN [126].

Structural changes in diabetic nephropathy include mesangial expansion, the loss of podocytes, the thickening of the glomerular basement membrane, and the formation of Kimmelstiel–Wilson nodules, pathological hallmarks of this condition [133]. The pathogenesis of DN is multifactorial, involving an intricate process where molecular, cellular, and physiological mechanisms converge, ultimately leading to chronic kidney disease in individuals with diabetes [134]. While diabetic nephropathy and diabetic retinopathy are both microvascular complications of diabetes, this response focuses on the mechanisms pertinent to DN. As DN involves altered blood flow and filtration within the kidney structures, these mechanisms cause damage over time, reducing kidney function [55,134]. The risk factors for DN include genetic predispositions, such as the Haptoglobin 2-2 phenotype, and it is known that around forty percent of people with diabetes will develop microvascular and macrovascular complications, which include not only DN but also diabetic retinopathy and vascular diseases such as coronary artery disease and stroke [126]. Diabetic nephropathy remains a significant concern for public health due to its association with increased morbidity and mortality in individuals with diabetes. Moreover, the COVID-19 pandemic has shown that diabetic patients with kidney disease are at an increased risk of hospitalization and premature death [135,136,137].

Interventions have evolved, with the Diabetes Control and Complications (DCCT) Research Group among those showing the benefits of stringent glucose control in reducing the risk of DN [138]. Ongoing research builds upon our understanding of this disease to improve treatment options [139].

### 3.6. Treatment and Current Advances in Diabetic Nephropathy

The current treatment of diabetic nephropathy includes tight glycemic control and management of blood pressure, with a preference for renin–angiotensin system (RAS) inhibitors. Specifically, angiotensin-converting enzyme inhibitors and angiotensin receptor blockers are essential as they help reduce proteinuria and slow progression to ESRD [140]. Additionally, the recent introduction of Sodium-glucose cotransporter 2 (SGLT2) inhibitors, such as dapagliflozin, has shown promise in treating patients with advanced diabetic kidney disease because of their glucose-lowering effects and benefits on renal outcomes [141]. Diverse therapeutic modalities are considered standard and emerging approaches, incorporating not only SGLT2 inhibitors but also aldosterone antagonists, endothelin receptor antagonists, and novel anti-fibrotic agents. These emerging treatments focus on different aspects of diabetic nephropathy progression, including fibrosis, oxidative stress, and inflammation [55,141]. Standard management practices also stress multifactorial interventions, targeting risk factors such as dyslipidemia, smoking, and obesity to complement pharmacotherapy. While tight glycemic control remains a cornerstone of diabetic nephropathy management, evolving research expands treatment possibilities, offering hope for improved renal prognoses in diabetic patients. It is important to personalize treatment plans and adjust them based on the patient’s clinical response and tolerance to therapies.

Various approaches are being explored for the treatment of diabetic kidney nephropathy. Oxidative stress is a significant contributor to the progression of diabetic kidney disease (DKD). The NRF2/KEAP1/ARE pathway regulates antioxidant response (ARE) activation and is an endogenous antioxidant pathway crucial in protecting against oxidative stress [142]. Oxidative stress has been identified as a critical target for therapeutic interventions in DKD, with various approaches aiming to reduce the excessive formation of ROS [136,137,143]. Studies have also investigated the potential of targeted antioxidant therapies, such as natural antioxidants or substances that activate the NRF2/KEAP1/ARE pathway, in treating DKD.

The potential therapeutic approaches that target DKD via oxidative stress include sulforaphane, resveratrol, allicin, grape seed proanthocyanidins, rutin, icariin, astaxanthin, coenzyme Q10, sodium butyrate, AB38b, DPHC, berberine, broussonetia kazinoki Siebold fruit extract, Nrf2 activators, oleander stem and root standardized extracts, genistein, sitagliptin, curcumin, bardoxolone methyl, isothiocyanates (ITCs) and the prominent representatives (sulforaphane and moringa isothiocyanate), cinnamic aldehyde, resveratrol, Eucommia ulmoides, other polyphenols, antioxidant compounds targeting mitophagy (CoQ10, MitoQ, and Astragaloside II), astaxanthin, drugs with NRF2 activity (fenofibrate, mycophenolate mofetil, and minocycline), and less-known NRF2 activators (sodium butyrate, AB38b, tetrandrine, and DPHC) [142]. These approaches aim to reduce oxidative stress and improve kidney function in DKD patients. Further research is needed to understand their efficacy and safety in thoroughly treating DKD.

Several clinical trials have shown the renoprotective effect of vitamin D treatment in patients with diabetic kidney disease [144]. However, there is a lack of consensus on the efficacy of vitamin D in treating diabetic kidney disease.

Other treatment approaches have targeted the renin–angiotensin–aldosterone system (RAAS), which helps regulate blood pressure and fluid balance in the kidneys. Agents targeting the RAAS pathway, such as angiotensin-converting enzyme inhibitors, angiotensin II receptor blockers, and renin inhibitors, are effective in slowing the progression of diabetic nephropathy [145]. Other possible future therapies that have shown promise in research studies include SGLT2 inhibitors, aldosterone antagonists, endothelin receptor antagonists, and novel anti-fibrotic agents [146].

LncRNAs have also been implicated in the development and progression of DN, which is a common complication of diabetes. They are involved in mesangial cell proliferation, fibrosis, inflammatory responses, and extracellular matrix accumulation in the glomeruli. Several lncRNAs are dysregulated in DN [79]. CYP4B1-PS1-001 is significantly downregulated in early diabetic nephropathy. Its downregulation has increased mesangial cell proliferation and fibrosis in vitro and in vivo [79]. NEAT1 (nuclear enriched abundant transcript 1) is significantly upregulated in the renal tissue of diabetic animal models and mesangial cells [147,148]. Its upregulation has been implicated in the pathogenesis of DN, particularly in glomerular injury and inflammation [149]. PVT1 (plasmacytoma variant translocation 1) has been identified as a candidate gene for end-stage renal disease in T2DM [150]. Its upregulation has been associated with the excessive accumulation of extracellular matrix in the glomeruli, a hallmark of DN [79].

TUG1 (taurine upregulated gene 1) is significantly repressed in the podocytes of diabetic mice. Its downregulation has been linked to podocyte injury and dysfunction, which contribute to the development of DN. MALAT1 (metastasis-associated lung adenocarcinoma transcript 1) is remarkably upregulated in the kidney cortices of diabetic mice with glomerular podocyte impairment and proteinuria. Its upregulation has been associated with increased vascular leakage and inflammatory cytokine expression in the diabetic retina. These lncRNAs, among others, have been implicated in the pathogenesis of DN and are potential targets for further research and therapeutic interventions [79].

According to recent research, nanotechnology-based interventions, such as nanocarriers targeting podocytes, may provide a future therapeutic option for targeting kidney diseases, including diabetic nephropathy. Nanotechnological approaches for targeting diabetic nephropathy involve using nanoparticles and other nanoscale materials to precisely deliver therapeutic agents to the kidneys and target the underlying molecular mechanisms of the disease. These approaches aim to improve drug efficacy, reduce side effects, and enhance the overall treatment outcomes for diabetic nephropathy. Some specific nanotechnological approaches for targeting diabetic nephropathy include prodrug nanocarriers, macromolecular-based nanocarriers, liposomes, nanoparticle sensors, and gene delivery systems [151].

Prodrugs are inactive drugs converted into an active form by enzymes or metabolism. They can be designed to specifically target the kidneys and release the active drug at the site of action. These prodrugs can be modified with specific moieties, such as amino acids, folate, or sugar, which interact with renal tubular receptors for targeted uptake [152]. Macromolecular carriers, such as low-molecular-weight proteins (LMWPs), can deliver kidney drugs. LMWPs, such as insulin, lysozymes, or light-chain immunoglobulins, are reabsorbed in the renal tubules after filtration at the glomerulus. By conjugating drugs to LMWPs, the drug can be precisely delivered to the kidneys and targeted to the desired cells or tissues [153]. Liposomes are lipid-based vesicles that can encapsulate drugs and deliver them to specific target sites. Liposomes can be modified with targeting ligands, such as peptides or antibodies, to enhance their specificity for renal cells or tissues. Liposomes can also reduce the nephrotoxicity of certain drugs by encapsulating them and delivering them directly to the kidneys [153,154]. Nanoparticles can be designed as sensors to monitor the progression of diabetic nephropathy [151]. These nanoparticles can detect specific biomarkers or changes in cellular activity associated with the disease. By tracking these changes, nanoparticle sensors can provide valuable information for disease monitoring and treatment evaluation. Nanoparticles can also be used as carriers for gene therapy in diabetic nephropathy [155,156]. They can deliver therapeutic genes to target kidney cells, promoting tissue repair and regeneration. Gene delivery systems based on nanoparticles offer the potential for the precise and controlled delivery of therapeutic genes to the affected renal cells.

These nanotechnological approaches for targeting diabetic nephropathy aim to overcome the limitations of traditional therapies by improving drug delivery, enhancing therapeutic efficacy, and reducing side effects. They hold promise for developing more effective and personalized treatments for diabetic nephropathy.

## 4. Impact of Glucose-Lowering Drugs on Microvascular Complications

Studies like ADVANCE (Action in Diabetes and Vascular Disease: Preterax and Diamicron Modified Release Controlled Evaluation), VADT (Veterans Administration Diabetes Trial), ACCORD (The Action to Control Cardiovascular Risk in Diabetes), ADDITION (the Anglo-Danish-Dutch Study of Intensive Treatment in People with Screen-Detected Diabetes), Steno-2, and the United Kingdom Prospective Diabetes Study have shown positive impacts on microvascular endpoints with intensive therapy, including a delay in disease progression and reduced risk of the composite cardiovascular event [157,158]. The microvascular outcomes of the drugs used in these trials are the reduction in microalbuminuria and macroalbuminuria, improvement in peripheral neuropathy, beneficial effects on nephropathy, reduction in the incidence/progression of retinopathy, and decrease in microvascular endpoints with tight blood pressure control. No consistent significant differences in microvascular complications between the intensive and standard therapy arms in the mentioned trials are observed. These studies provide insights into the effects of different treatment approaches on these outcomes in individuals with diabetes.

Many positive outcomes related to cardiorenal function due to the use of SGLT-2 inhibitors, including improvements in heart failure, blood pressure, cardioprotective effects in patients with ASCVD, nephroprotection, renal protection, reduction in the loss of eGFR, prevention of kidney failure, and reduction in the risk of cardiovascular events and mortality in both diabetic and non-diabetic patients, are reported. These outcomes demonstrate the significant impact of SGLT-2 inhibitors on cardiorenal health, emphasizing benefits in heart failure, kidney health, and overall cardiovascular outcomes. Among SGLT2 inhibitors, empagliflozin has demonstrated favorable renal outcomes in patients with renal impairment and diabetes [159,160].

GLP-1 analogs can also be used in significant renal impairment. Liraglutide and dulaglutide are licensed for use in eGFR down to 15 mL/min. They are well tolerated and do not need dose adjustment in severe renal impairment. Canagliflozin (an SGLT2 inhibitor) was associated with an increased risk of amputation, while liraglutide (a GLP-1 analog) reduced the incidence of amputation. Subcutaneous semaglutide was linked to an increased risk of diabetic retinopathy. Additionally, the confidence in effect estimates for these outcomes was reported to be low [160,161].

Liraglutide has been shown to improve coronary microvascular function, protect pancreatic islet endothelial cells, reduce oxidative stress, and enhance endothelial function. It also protects against hypoxia/reoxygenation injury and the potential benefits in obesity and prediabetes. Liraglutide acts as a nephroprotective agent, reducing the occurrence and progression of nephropathy. The drug has beneficial effects on microvascular outcomes by reducing hyperglycemia-induced endothelial dysfunction and preventing endothelial cell apoptosis [162].

DPP-4 inhibitors like vildagliptin, sitagliptin, and saxagliptin have shown effectiveness in managing type 2 diabetes, improving glycemic control, and impacting microvascular aspects such as renal and liver function. These inhibitors have a low risk of hypoglycemia, do not cause weight gain, and have been studied in patients with renal and liver impairments [160].
